# Preparation of an elderberry anthocyanin film and fresh-keeping effect of its application on fresh shrimps

**DOI:** 10.1371/journal.pone.0290650

**Published:** 2023-11-29

**Authors:** Na Guo, Miaomiao Song, Wei Liu, Fangyan Zhang, Guilan Zhu

**Affiliations:** 1 Department of Biological and Food Engineering, Hefei Normal University, Hefei, Anhui, China; 2 School of Food and Biological Engineering, Hefei University of Technology, Hefei, Anhui, China; Shiraz University, ISLAMIC REPUBLIC OF IRAN

## Abstract

A smart packaging film was developed employing the pH-indicating activity of elderberry anthocyanins to solve the problem of refrigerated food freshness monitoring. The effect of elderberry anthocyanins on the properties of gellan gum, gelatin composite films and preservation of fresh shrimp as an indicator of freshness was investigated. The results showed that the elderberry anthocyanin-gellan gum/gelatin film had improved on film thickness (7.8×10^−2^ mm), TS (tensile strength) (14.57×10^3^ MPa), WVP (water vapor permeability) (36.96×10^−8^ g/m·s·Pa), and a reduced EAB (elongation at break) (17.92%), and water solubility (water-soluble time of 60.5 s). SEM (scanning electron microscopy) and FTIR (infrared spectrum analysis) showed excellent compatibility between its components. Moreover, the elderberry anthocyanin film exhibited good mechanical properties and pH indication effects. Therefore, the film can be considered suitable for maintaining the quality of fresh shrimp. The results could provide a reference for research and development into new active intelligent packaging films.

## 1. Introduction

Proteins break down while storing foods such as meat, shrimp, and crabs, producing total volatile basic nitrogen (TVB-N) such as ammonia and low-grade amines, which can affect the pH of the foods [1]. Therefore, measuring the change in pH is a valuable way to determine the degree of degradation of food, and the food pH monitoring system based on this concept is a very intelligent and practical detection technology. The newly reported pH-color indicator can visually transmit real-time food quality information, and the device is simple, efficient, and widely utilized for monitoring frozen and chilled food storage at low temperatures [2–4].

Food packaging films play an important role in food production and processing because they protect food from numerous risks during manufacture and transportation while extending its shelf life [5–8]. The edible components used in the current study of indicating smart packaging films, such as carrageenan, gellan gum, and gelatin, can be molded into composite packaging films with improved structural qualities, freshness preservation, and pH-color indicating effects. There are packaging materials formed by a composite of different materials that, due to interactions between the molecules of the film components, can form a protective film with good barrier properties on the surface, with the role of moisture and reducing drying of the food’s surface, and so on [9]. Adding antioxidant polyphenols to edible films can increase polyphenol stability and improve the antioxidant capabilities of composite films [1, 10–12]. Furthermore, the composite film can limit the growth of harmful bacteria and spoil microorganisms.

Gellan gum is a polysaccharide derived from transparent microorganisms that are simple to combine with other colloids. Films made from gellan gum have strong tensile strength and good barrier characteristics and are easily miscible with other film-forming ingredients [13–15]. Studies have shown that the compound of gellan gum and polyphenols can improve the bioactivity of polyphenols [16–18]. Gelatin is a biodegradable and biocompatible protein from animal bone, skin, and other parts. The gelatine film has good water-blocking and light and oxygen escape-blocking capabilities, but its film-forming and mechanical qualities are poor, and its performance has to be improved [19, 20].

Elderberry anthocyanin is a naturally occurring antioxidant and water-soluble pigment whose primary structural component is the 2-phenyl-benzopyran cation. It is used extensively in China to cure fracture and joint problems as well as to increase bone mass and bone strength [21, 22]. The color of anthocyanin changes with structure, being red under acidic conditions, purple under neutral conditions, tawny under alkaline conditions, and can indicate pH changes in food [23].

Numerous studies have been done on active film of polyphenols, such as anthocyanin, curcumin which not only the antioxidant properties of the plastic wrap were enhanced, but also the pH value of the fresh food was significantly responsive. The present study aimed to combine the properties of gellan gum and gelatin to produce a composite film with strengthened mechanical properties. Elderberry anthocyanin was added to improve the antioxidant and antibacterial properties of the preservative film and to provide a means of judging food freshness. The study will thus present a technology for monitoring freshness, thus providing a reference for developing intelligent packaging films.

## 2. Materials and methods

### 2.1 Materials

Low acyl gellan gum (with a purity of 97.3% and gel strength > 1129g/cm^2^ with 0.5%solution, food grade) and bovine bone gelatin (with a purity of 85.8% and jelly strength is greater than 126 Bloom/g, food grade) were purchased from Henan Minrui Biotechnology Co., Ltd. (Zhengzhou, China); elderberry anthocyanin (food grade, concentration of elderberry anthocyanin was calculated to be 20.04 mg/g) from Qufu Shengjiade Biotechnology Co., Ltd. (Qufu, China); and reagents (analytical grade) from China National Medicines Corporation Ltd. (Beijing, China).

Fresh shrimps are common shrimps raised and purchased at local markets in Hefei market (Anhui, China).

### 2.2 Preparation and testing

#### 2.2.1 Preparation of blended films

The gellan gum and gelatin were weighed and then dissolved in 60 mL of distilled water to prepare a 2% blended film solution. The solution was heated in a water bath at 60°C, mixed using a glass rod, and stirred well. After being left to cool down, 0.5 mL of elderberry anthocyanin solution (0.2 mg/mL) and 0.5 mL of plasticizer glycerol were added to the solution, stirred well using a glass rod then stored in a refrigerator at 4°C for 24 h to ventilate. The film was coated evenly on a metal tray using the casting method, placed in an oven at 45°C for 48 h then left to equilibrate for 24 h. Before testing its properties, the film was peeled off and stored in the dark.

In the present study, weight ratios of gellan gum and gelatin of 100:0, 80:20, and 0:100 were used to prepare gellan gum film, gelatin film, gellan gum/gelatin film, gellan gum-elderberry anthocyanin film, gelatin-elderberry anthocyanin film, and the gellan gum/gelatin-elderberry anthocyanin film and labeled GG, GL, GL/GG, GG+SWA, GL+SWA, and GL/GG+SWA, respectively.

#### 2.2.2 Determination of the thickness of the blended films

Five points were randomly selected on the film being tested, and the thickness was measured using a micrometer (accurate to 0.001 mm). The average of the five measurements was taken as the thickness.

#### 2.2.3 Determination of the tensile strength and elongation at break of the blended films

The film was cut into 2.0×8.0 cm strips, clamped in the upper and lower jigs. A texture analyzer (TA-XT plus, Stabie Micro Systems, UK) was used to measure the mechanical properties of the film, with a tensile load of 20 g, the distance between the upper and lower clips of 25 mm, and a stretching rate of 0.2 mm/s.

The tensile strength (TS) refers to the ratio of the maximum tensile load before rupturing to the product of the width and thickness of the film under the action of the axial tensile force. TS was calculated using Eq (1).

$$TS=\frac{F}{L\times S}$$ (1)

Where: TS is the tensile strength (MPa); F, the axial tensile force (N); L, the film width (mm); and S, the film thickness (mm).

The Elongation at break (EAB) refers to the rate of change of the length of the film when the film is broken. EAB was calculated using Eq (2).

$$EAB=\frac{l-l_{1}}{l_{1}}\times 100\%$$ (2)

Where: EAB is the elongation at break (%); l_1_, the length of the sample before tensioning (mm); and l, the length of the sample after tensioning (mm).

#### 2.2.4 Determination of the water vapor permeability of the blended films

Conical flasks of the same size and specification were used. Anhydrous CaCl_2_ was thoroughly fully dried in an oven to constant weight then a 10-g portion was placed in a conical flask which was sealed with the film to be tested. The conical flask with its contents was then weighed then placed in a balancer which maintained a certain vapor pressure on both sides of the sample. The weight gain of the conical flask per unit time was measured then the water vapor permeability (WVP) was calculated using Eq (3).

$$WVP=\frac{q\times d}{t\times s\times \Delta p}$$ (3)

Where: WVP is the water vapor permeability (g/m·s·Pa); q/t, the average weight gain per unit time of the flask during steady penetration (g/d); d, the film thickness (m); s, the area of the film tested (m^2^); and Δp, the vapor pressure difference between the two sides of the film (Pa).

#### 2.2.5 Water solubility of the blended films

The prepared film was cut into pieces with size of 3 cm × 3 cm, put it into a beaker containing 60 mL of distilled water. The beaker was preheated in a 60°C constant temperature water bath in advance. Then the film was put into the beaker and timing started at the same time. The time required for complete dissolution of the film was recorded.

#### 2.2.6 Response of anthocyanin solution to pH

The anthocyanin solution (0.2 mg/mL) was put in buffers with different pH values, mixed well then left to stand for 3 min. Color changes were observed. An ultraviolet-visible spectrophotometer (UV-1800, Shimadzu Corp., Kyoto, Japan) was used to detect the UV absorption spectrum of the anthocyanin at pH values between 2 and 12, using a scanning wavelength from 300 to 800 nm.

#### 2.2.7 Infrared spectrum of the blended films

Using a Fourier transform infrared spectrometer (FT-IR) (Nicolet-6700, Thermo Scientific Nicolet, Madison, WI, USA), the sample was scanned 100 times in the wavelength range of 4000-400 cm^-1^ with a KRS-5 ATR probe at a resolution of 4 cm^-1^, and the infrared spectrum of the sample was recorded.

#### 2.2.8 Thermogravity of the blended films

The stability of the film sample was tested using a thermal analyzer (STA449F5, NETZSCH, Selb, Germany). A sample of film (8 mg) was placed in a crucible. The scanning temperature was 30-600°C and the heating rate, 10°C/min. The operation was conducted in flowing N_2_ and the film was kept dry.

#### 2.2.9 Scanning electron microscopy (SEM) observations of the blended films

The surface and cross-section of the film sample were observed using a scanning electron microscope (EVOMA-15, Carl Zeiss Microimaging GmbH, Göttingen, Germany). After drying at 45°C for 24 h, the cross-section, and surface of the film were sprayed with gold then the film microstructure was observed using an accelerating voltage of 1.0 KV.

#### 2.2.10 Coating fresh shrimps with film for the preservation studies

Fresh, whole, large shrimps were chosen, cleaned, and dried, then weighed in groups. A part of the shrimp was placed in triangular flasks, wrapped tightly with film, and finally stored in a refrigerator at 4°C. The color change of the film was observed during the storage phase. Another part of the shrimps was immersed in the film solution for 5 min and dried, then stored in a 4°C refrigerator to determine the shrimps’ color change and physicochemical properties.

#### 2.2.11 Determination of TVB-N

The TVB-N of the shrimps was determined according to method of Minmin Chen with auto-Kjeldahl analyse [24].

#### 2.2.12 Determination of fresh shrimp texture

The texture of fresh shrimps was determined using a texture meter (TA-XT plus, Stabie Micro Systems, UK). Parameters was set as: the hardness and elasticity of fresh shrimps were determined using the TPA mode with an edible P100 probe and a speed of 1 mm/s.

### 2.3 Data analysis

Experiments data were analyzed using analysis of variance (ANOVA) with a significant level of α = 0.05, SPSS 24.0 software and drawing with Origin. Data were expressed as mean ± standard deviation.

## 3. Results and discussion

### 3.1 Analysis of film thickness and mechanical properties

The tensile strength of the gelatin film was lower than that of the gellan gum film, according to Table 1. On the other hand, the composite film’s TS was greatly increased, reaching 30.69×10^2^ MPa, indicating the presence of intermolecular forces between the gelatin and gellan gum that the polymer structure network produced had improved the TS [25]. This could be due to the interaction of the amino acid molecules in gelatin with the hydroxyl groups in gellan gum, which raises the molecular stiffness of the complex. The TS of the composite film with additional anthocyanin was 15.42×10^2^ Mpa, which was much higher than that of the pure gellan gum or gelatin films, perhaps due to enhanced tangling of intermolecular hydrogen bonds caused by the anthocyanin molecules.

**Table 1:** FT, TS, EAB and WVP of the films.

Sample	FT ×10^−2^(mm)	TS ×10^3^(MPa)	EAB (%)	WVP ×10^−8^(g/m·s·Pa)	Solution time (s)
GL	6.8±0.4^b^	1.28±0.15^c^	55.69±3.71^b^	10.55±0.64^c^	170.5±25.5^b^
GG	5.9±0.1^c^	8.71±1.07^b^	24.45±2.11^bc^	9.06±1.35^c^	36.5±0.5^c^
GL/GG	7.9±0.1^a^	5.46±1.30^b^	18.59±1.52^c^	23.95±3.25^b^	74.3±12.3 ^c^
GL+SWA	6.0±0.4^bc^	0.81±0.01^c^	48.10±0.41^a^	7.81±1.64^c^	255.5±15.5^a^
GG+SWA	6.3±0.1^b^	8.36±0.09^b^	29.10±0.98^b^	9.45±0.55^c^	47.5±4.5 ^c^
GL/GG+SWA	7.8±0.2^a^	14.57±3.19^a^	17.92±3.15^c^	36.96±0.80^a^	60.5±8.5 ^c^

Values are mean ± SD mean and the values are in same row with sharing same subscripts letter are not statistically significant (P<0.05).

All of the films with additional anthocyanins showed lower EBA, with gelatin having the greatest elongation at break. Gelatin has a lot of polar groups like -OH and -NH_2_, which easily combine with gellan gum to form intermolecular forces like hydrogen bonds. Furthermore, anthocyanins are hydrophilic, forming more hydrogen bonds with water molecules, making composite films stiffer and brittle, and lowering EBA.

### 3.2 Water vapor permeability (WVP) and water solubility of the films

The ability of a film to withstand external moisture is determined by its water vapor permeability (WVP). Food packaging materials are being developed to limit water flow to reduce food deterioration, which mandates using a low WVP material. The WVP (Table 1) of the gelatin/gellan gum composite film increased because both gelatin and gellan gum are hydrophilic. As a result, water molecules are more likely to pass through the film, increasing the intermolecular free volume and, as a result, increasing water vapor permeability [26]. The WVP of the elderberry anthocyanin-gellan gum/gelatin film is the greatest, reaching 34.20×10^−8^ g/m·s·Pa. This change may be because there are many hydrophilic phenolic hydroxyl groups in elderberry anthocyanin, allowing water molecules to pass through the film more easily, thus increasing the WVP of the composite film.

Another important property of edible films is their water solubility, which reflects the material’s affinity for water [27]. The water solubility of the gellan gum film was lower than that of the gelatin film from Table 1. This is because gellan gum has a higher hydrophilicity and a lower capacity to withstand moisture. The water solubility of the gelatin and gellan gum composite film increased, owing primarily to gelatin’s insolubility in cold water. The water solubility of the film was influenced by elderberry anthocyanin, which may interact with the gelatin and gellan gum and become entrenched between the film molecules, lowering water and component cross-linking. After swelling from water absorption, it created a stable network structure, resulting in a longer water-soluble time.

### 3.3 Response of elderberry anthocyanin and films to pH

**Fig 1:**
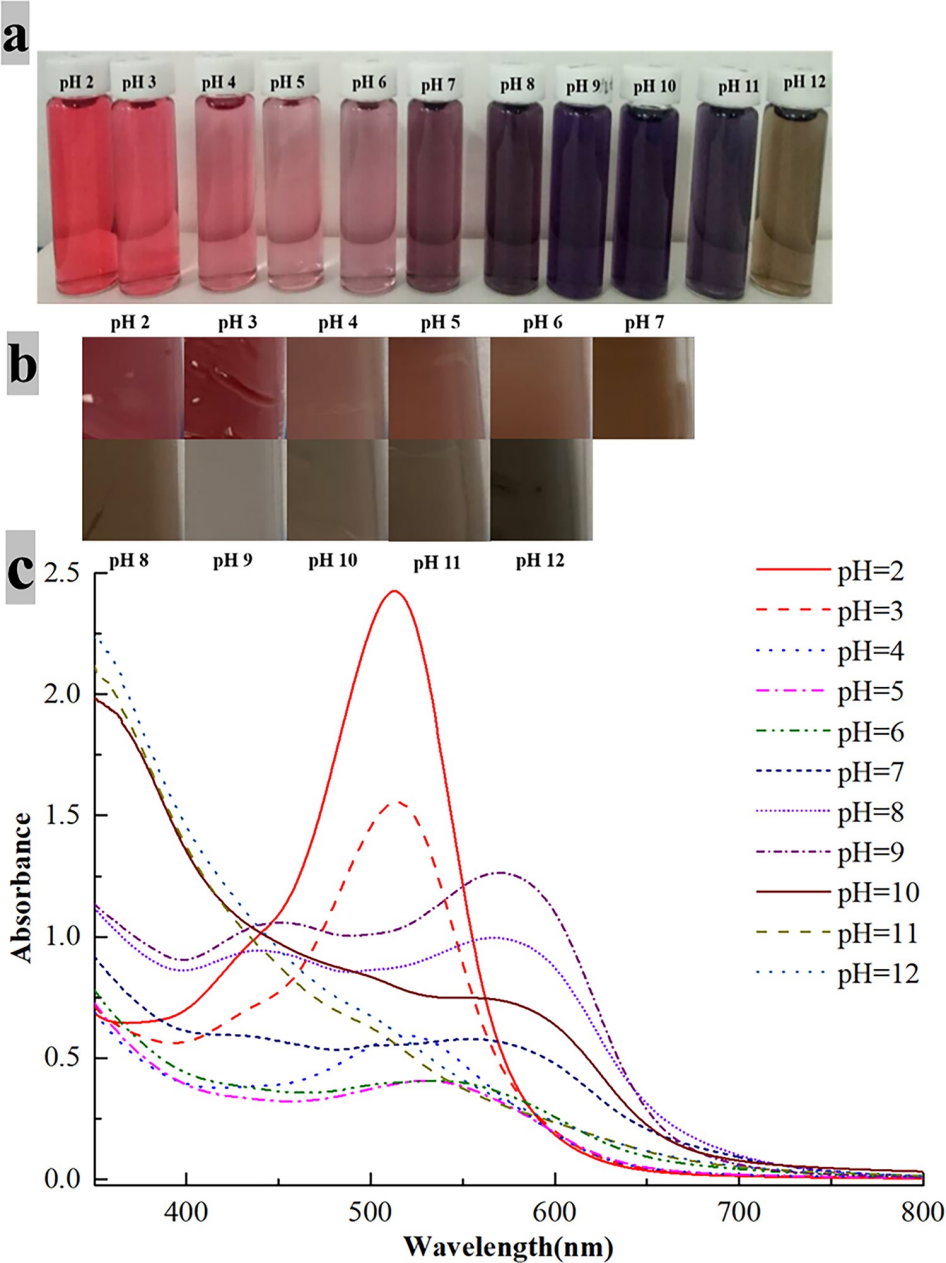
Changes in color of EA solutions and films under different pH conditions.

The color variations in elderberry anthocyanin and films at various pH values are shown in Fig 1. The color shifted from red to pink, lavender to purple, and dark purple to yellow-brown as the pH went from 2 to 12. This variation is mostly due to the structural transition of cornflower anthocyanin-3-O-glucoside, the major component of elderberry anthocyanins, under acidic and alkaline circumstances, such as quinone bases and methanolic pseudo-bases, which results in a color shift.

According to the UV scanning figure, the varied architectures of elderberry anthocyanin influenced the maximum absorption wavelength and absorbance. The absorbance value fell as the pH increased, the maximum absorption peak wavelength shifted to 540 nm, and the hue changed from red to pink. The color was purple in a pH 6–9 buffer but darkened as the pH increased, with maximum absorption at 570 nm. The wavelength of the maximum absorption peak increased with pH, as did the maximum absorbance, and the hue changed from pink to purple. The color was dark purple at pH 10, and the absorbance was substantially lower.

### 3.4 Analysis of the infrared spectrum of the blended films

**Fig 2:**
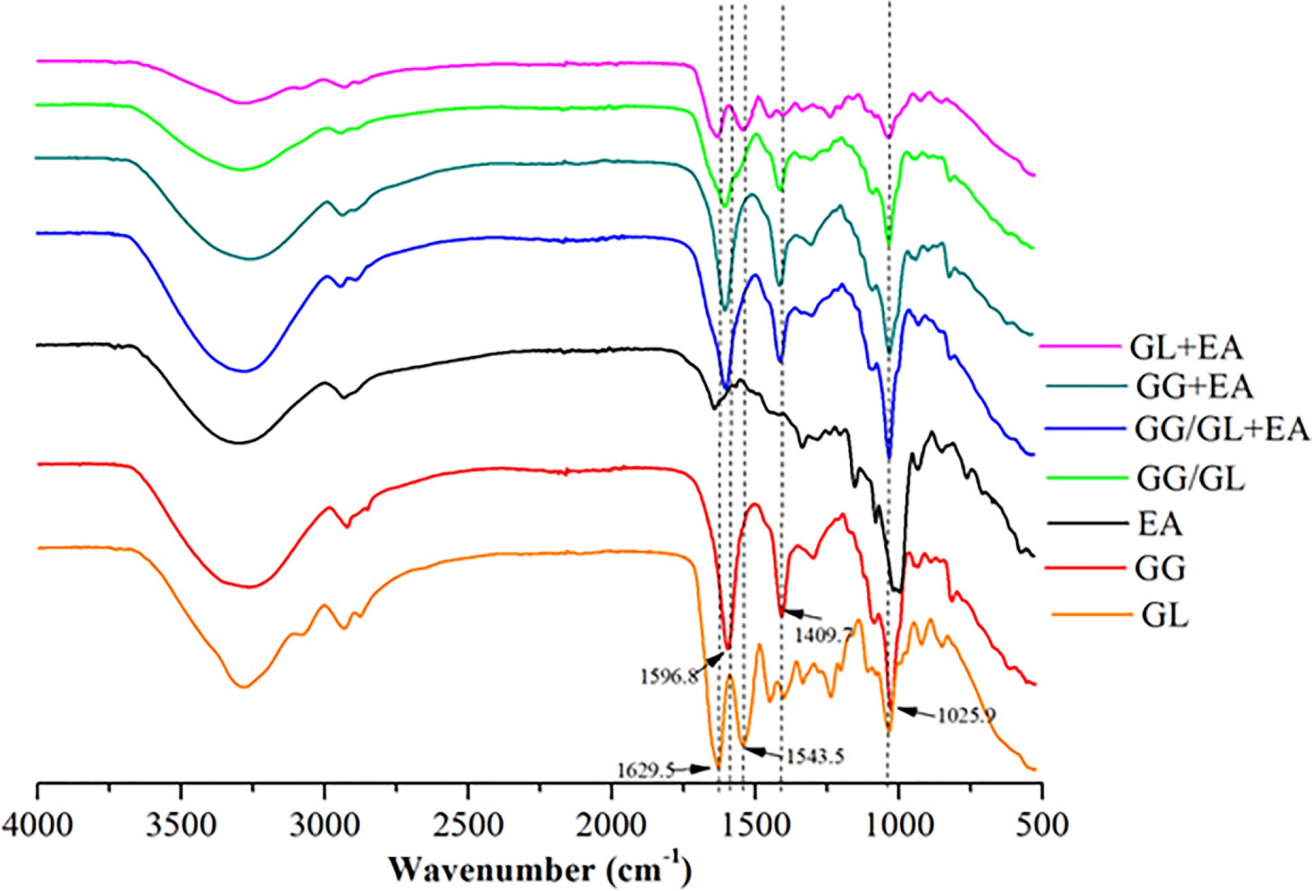
FTIR spectra of the films.

The intermolecular forces between different components determine the mechanical properties, water content and other important factors of the anthocyanin film [28]. The vibration and stretching patterns of distinct groups in the molecules of the composite film can be identified using infrared spectroscopy to explore the intermolecular interaction between the constituents [11]. In the gelatin film, the vibration absorption peaks of amide bond I and structural lactam bond II were detected at 1629.5 cm^-1^ and 1543.5 cm^-1^, respectively (Fig 2). There were three distinct peaks in the gellan gum film: 1596.8 cm^-1^, 1409.7 cm^-1^, and 1025.9 cm^-1^. These were the symmetric and antisymmetric stretching vibrations of the COO- group and the COO-C group’s C-O stretching vibration. The absorption peaks of anthocyanin-containing gelatin and gellan gum films did not vary considerably.

Nonetheless, the typical absorption of the gellan gum and gelatin composite film changed drastically. The results also demonstrated that the gelatin and gellan gum film’s specific absorption peak with added anthocyanin did not differ significantly. The characteristic absorption of the gellan gum and gelatin composite film changed significantly: the amide bond I absorption peak shifted from 1629 cm^-1^ to 1600 cm^-1^, indicating an intermolecular interaction between the gelatin and the gellan gum. The absorption peak of amide bond II at 1543.5 cm^-1^ in the composite film faded, indicating that gelatin and gellan gum interacted covalently at the time. The antisymmetric stretching vibration of the COO- group had changed from 1409.7 cm^-1^ to 1407.7 cm^-1^, indicating that the gellan gum had reacted with the gelatin [29].

### 3.5 Analysis of scanning electron microscopy (SEM) observations of the blended films

**Fig 3:**
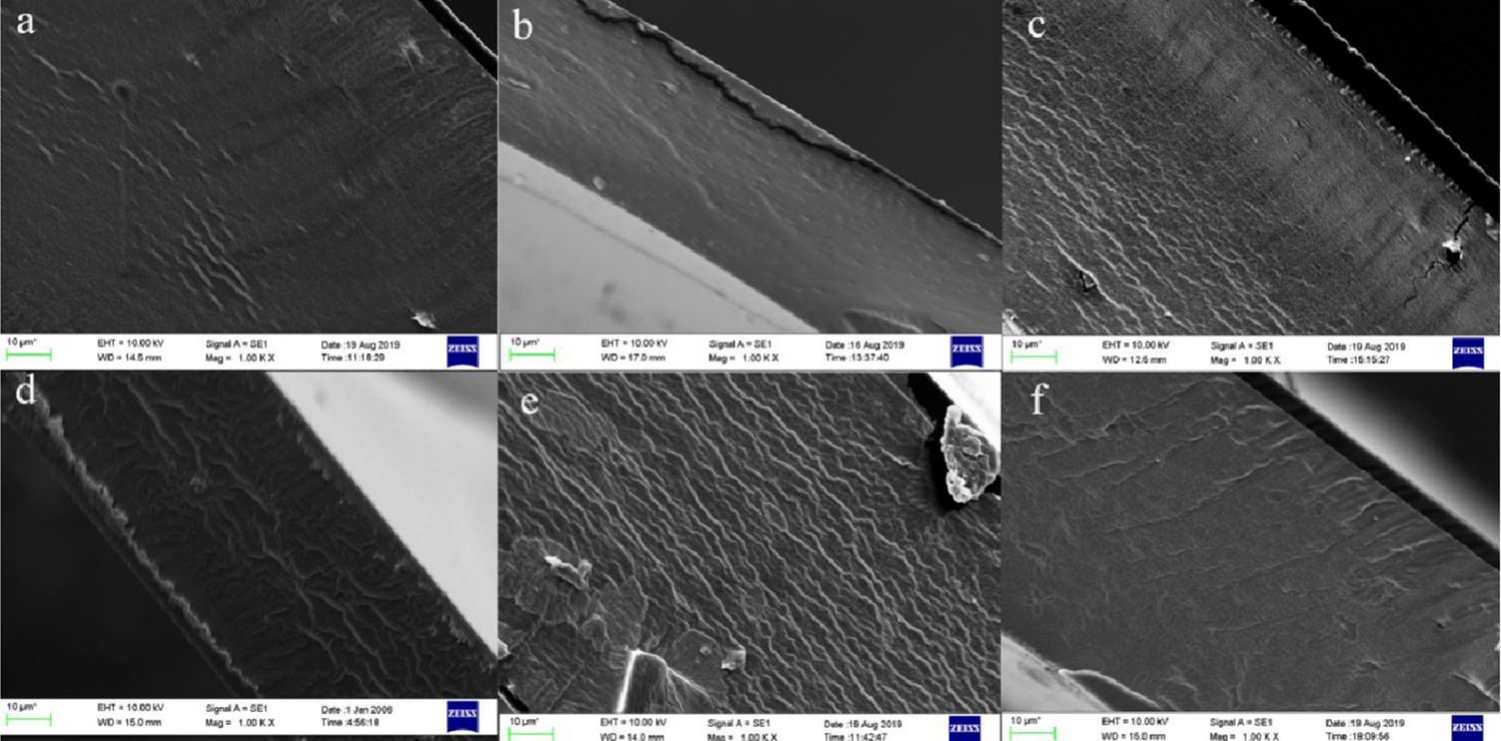
The cross-section morphology of films (a is GL film, b is GG film, c is GL+ EA film, d is GG+EA film, e is GG/GL film, f is GG/GE+EA film).

The compatibility of anthocyanin pigments with the film-forming matrix dictates whether anthocyanins can respond to variations in food freshness during storage [30]. The compatibility of materials can be reflected in SEM pictures, which indicates that the more uniform and smooth the film section, the better the compatibility between the film components. Cross-sections of the whole film (Fig 3) were devoid of bubbles or cracks, showing that the gellan gum, gelatin, and gellan gum/gelatin exhibited good film-forming compatibility [31]. The gellan gum/gelatin film had a homogeneous and tight cross-section with the addition of elderberry anthocyanin. The number of lines had decreased dramatically, indicating that the composite film had been evenly combined. This homogeneous integration was attributed to the high compatibility of gellan gum, gelatin, and elderberry anthocyanin molecules, as well as the hydrogen bonds generated between the elderberry anthocyanin and the film-forming substrate, which minimized intermolecular entanglement and smoothed the film structure.

### 3.6 Thermogravimetric analysis of the blended films

**Fig 4:**
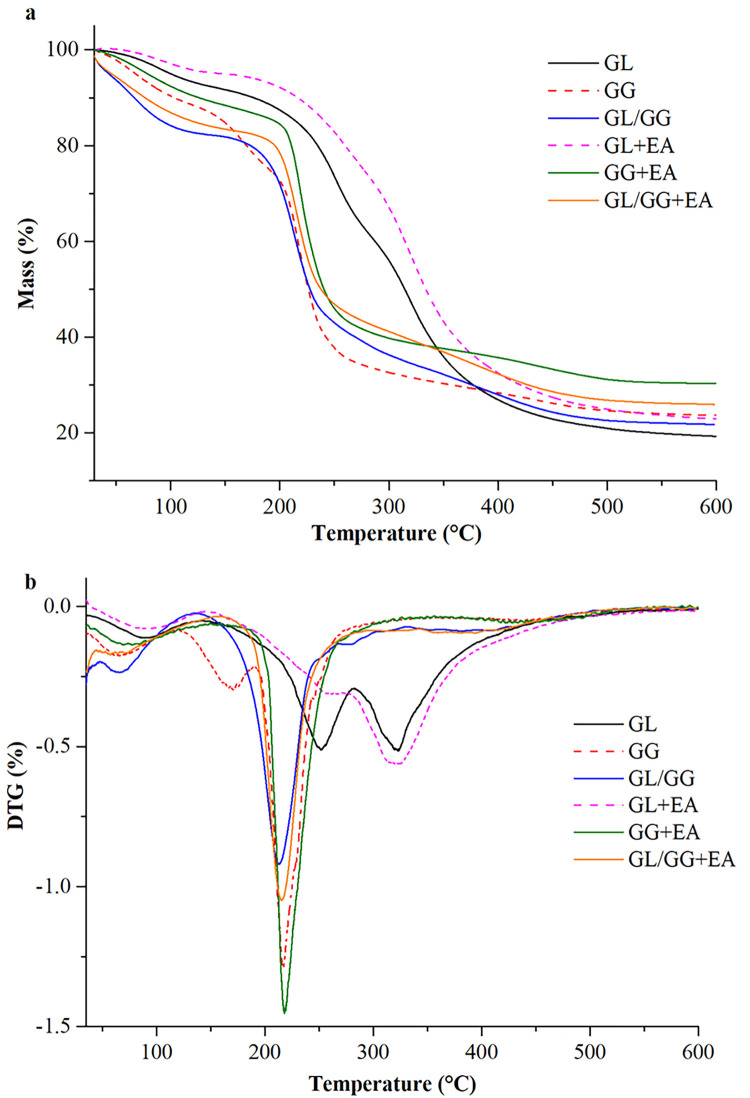
TG and DTG of the films.

The thermal stability of a packaging film is crucial in determining the thermal resistance of films during sterilization and microwave heating. The film lost mass in three stages, as shown in Fig 4. The first stage occurred between 50 and 120°C, presumably due to the evaporation of weakly bound free water in the film [32]. The second stage happened between 120 and 250°C, probably due to glycerol degradation. Moreover, the third stage started between 280 and 350°C, possibly due to the molecular disintegration of gellan gum, gelatin, and anthocyanin, as well as the decomposition of the microscopic polymeric structure. The DTG showed that anthocyanins contribute to the thermal stability of the composite films, with the gelatin-anthocyanin film being the most thermally stable. This may be due to the stronger intermolecular interactions between gelatin and anthocyanins, which require more thermal energy to degrade. The thermal stability of ternary composite film with added anthocyanins increased, although not as much as that of binary composite membranes with added anthocyanins.

### 3.7 Color changes during the preservation experiments of shrimps

**Fig 5:**
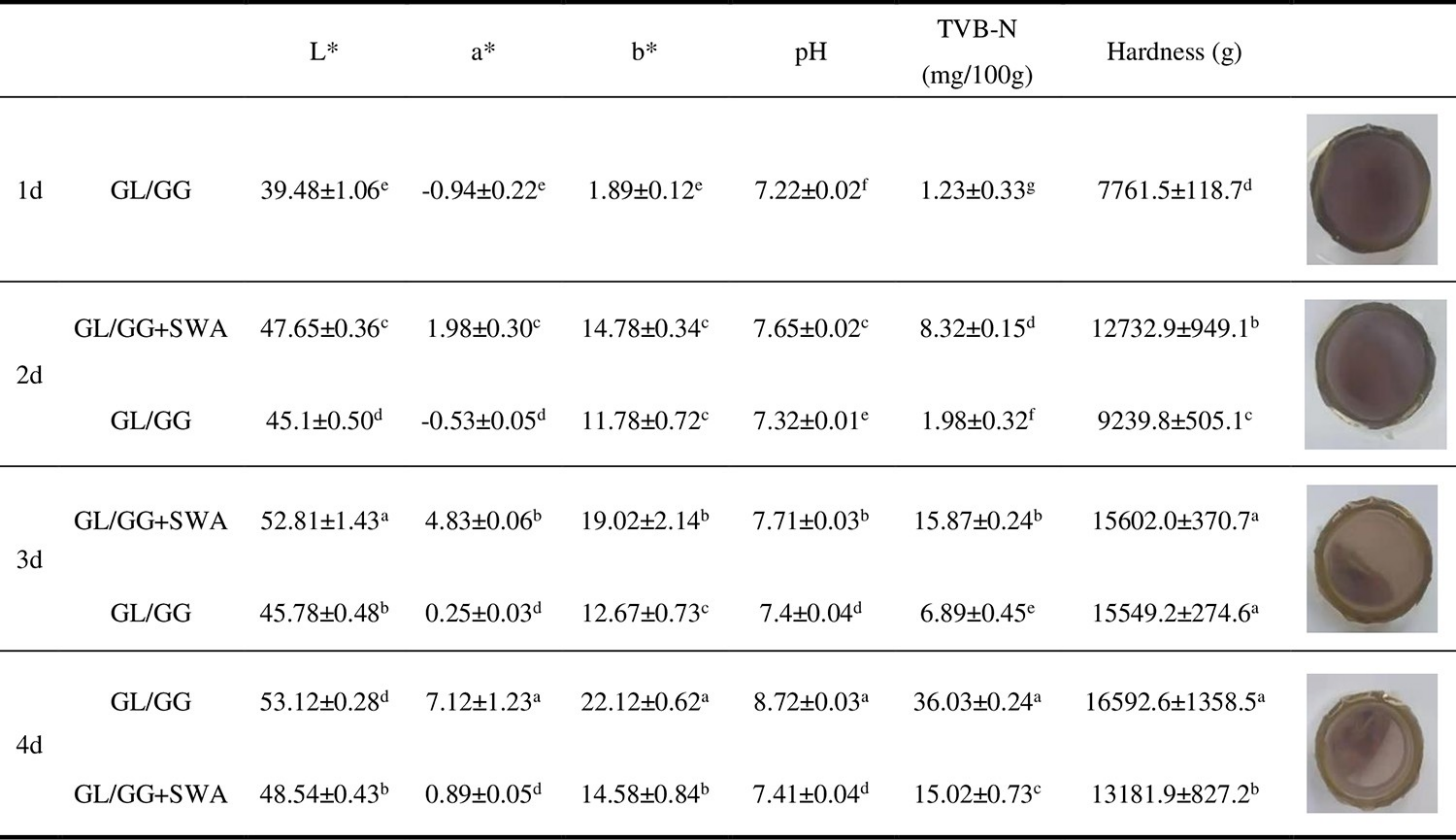
Changes in the color and physicochemical properties of fresh coated shrimps.

The astaxanthin in the shrimps was oxidized during chilling and preservation, changing its hue from blue-black to red. The shrimp in the control group turned red, as shown in Fig 5. The color shift in shrimp covered with anthocyanin film was less pronounced than in a control film. The color change of the shrimps covered with films containing 0.3 mg/mL of anthocyanins was relatively small, indicating that the anthocyanin films were effective in preserving freshness, possibly due to the antioxidant effect of elderberry anthocyanins, which prevented the fresh shrimps from deteriorating and changing color.

The investigation also found a substantial shift in the color of the cling film over time. The composite film’s color shift is obvious to the naked eye in the image. The color of the cling film changed from mauve to lighter on the first day, eventually exhibiting yellowish green, and the transparency of the film grew. The composite film, in other words, responds well to variations in environmental pH.

### 3.8 Changes in pH and TVB-N during storage experiment

The respiration cycle is disrupted when shrimp die, and the lactic acid produced in glycogen metabolism accumulates and lowers the body’s pH. Microorganisms in the body break down protein, producing alkaline chemicals such as volatile base nitrogen, and the pH steadily rises. As a result, the more spoiled the shrimp become, the higher the pH value of their body tissue.

According to Fig 5, the pH value of shrimps in the anthocyanin film-coated group changed less than in the non-anthocyanin film-coated group. The pH value of the coated film group was steady and modest, indicating that the coating anthocyanin film suppressed the growth and reproduction of microbes in the shrimp. The nitrogen concentration of fresh shrimp increased considerably in the non-coated group. Despite surpassing the Chinese national permissible limit of 30 mg/100 g of volatile base nitrogen in shrimp, the TVB-N value in the anthocyanin-coated group decreased slowly. It did not exceed the limit on the fourth day of chilled storage. The findings showed that the anthocyanin layer might limit protein degradation in vivo, retaining the freshness of fresh shrimp.

### 3.9 Changes in texture during chilled storage

Shrimp tissues are damaged by enzymes and microorganisms, which may alter the texture of the meat. The hardness of fresh prawns in uncoated and experimental groups gradually increased during storage (Fig 5). As can be seen from Fig 5, the hardness of fresh shrimp in the uncoated and experimental groups gradually increased during storage. In contrast, the film-coated group increased relatively slowly, suggesting that adding anthocyanins have a specific effect in maintaining the quality of shrimp during cold storage.

## 4. Conclusion

With the increased demand for food safety, the development of visual pH sensors for food packaging has received a lot of attention. The link between pH value variation and food freshness during storage due to protein breakdown or microbial growth requires more investigation.

In this study, an edible intelligent pH-sensitive films were prepared utilizing gellan gum, gelatin, and elderberry anthocyanins. The results demonstrated that the interactions of gellan gum, gelatin, and anthocyanin molecules had good compatibility and thermal stability. The composite film had higher TS and WVP than the simple film, and it decomposed quickly in water. The composite film was pH sensitive and might be employed as an active smart edible film for food preservation.
